# Supplementary material for: RIViT-seq enables systematic identification of regulons of transcriptional machineries
Source: Nat Commun. 2022 Jun 17;13:3502. doi: 10.1038/s41467-022-31191-w (PMC9205884; doi:10.1038/s41467-022-31191-w)
Supplement: Supplementary file 1 — Supplementary information [file 41467_2022_31191_MOESM1_ESM.pdf]

Supplementary Information

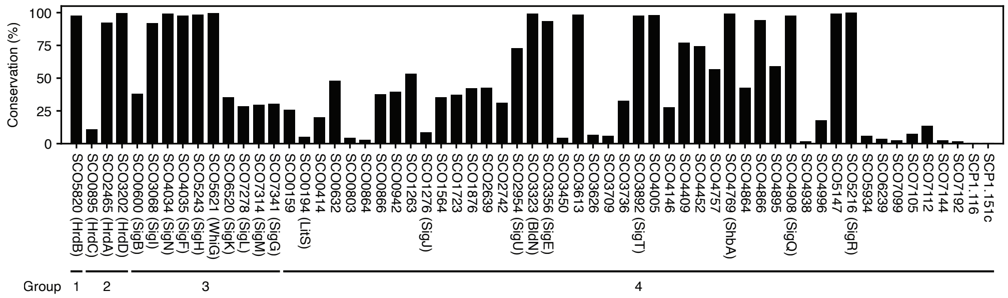

Supplementary Figure 1. Conservation of the *Streptomyces coelicolor* A3(2) sigma factors in streptomycetes and the group (or subfamily) assignment of sigma factors. The sigma factors with prefix "SCO" and "SCP" are encoded on the chromosome and the plasmids, respectively.

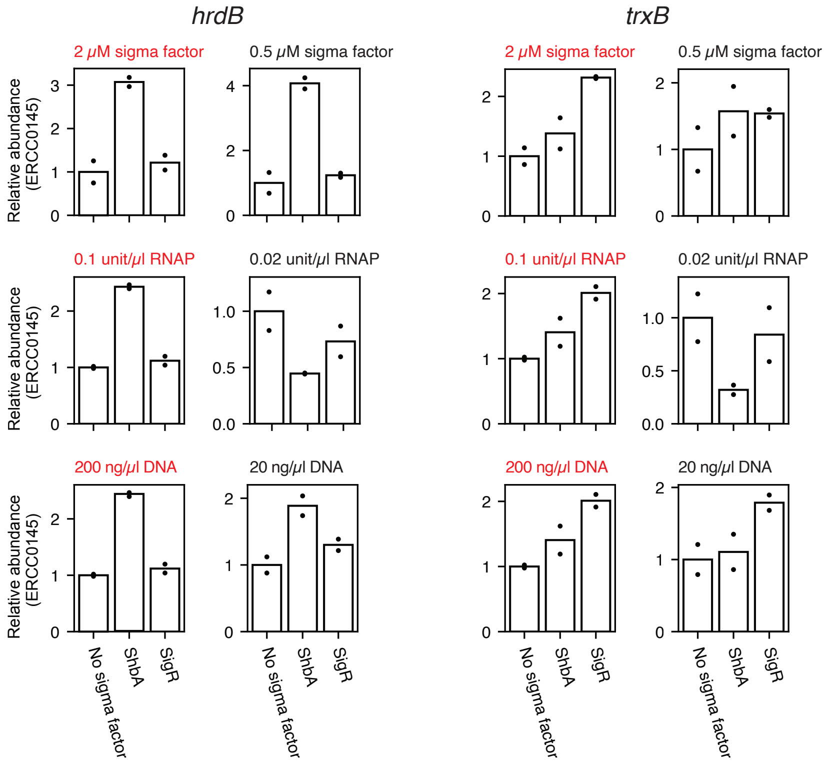

Supplementary Figure 2. Optimisation of the *in vitro* transcription assay with ShbA and SigR. The varying concentrations of the sigma factor, RNA polymerase core enzyme and genomic DNA were tested. The ERCC0145 was used as the normalisation control. Values are relative abundances of the *hrdB* and *trxB* transcripts of the samples with ShbA or SigR compared to the mean abundance of the "No sigma factor" samples. Error bars are standard deviations ($n = 2$ independent experiments). Conditions shown in red are those optimal for RIViT-seq.

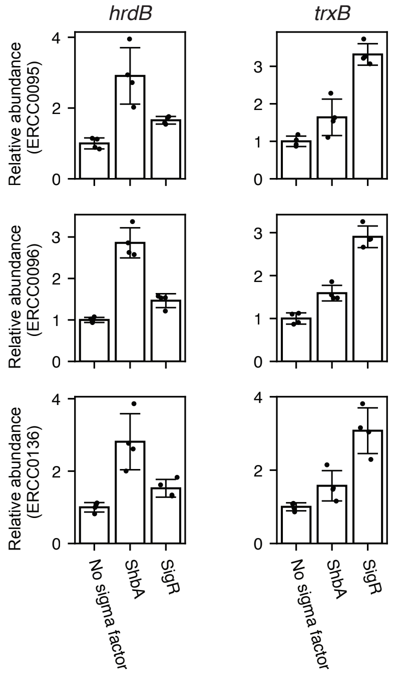

Supplementary Figure 3. Verification of the *in vitro* transcription assays with ShbA and SigR by quantitative RT-PCR. The relative quantities of the *hrdB* and *trxB* transcripts were analysed. The ERCC0095, ERCC0096 and ERCC0136 transcripts were used as the normalisation control. Values are relative abundances of the *hrdB* and *trxB* transcripts of the samples with ShbA or SigR compared to the mean abundance of the "No sigma factor" samples. Error bars are standard deviations (n = 4 independent experiments).

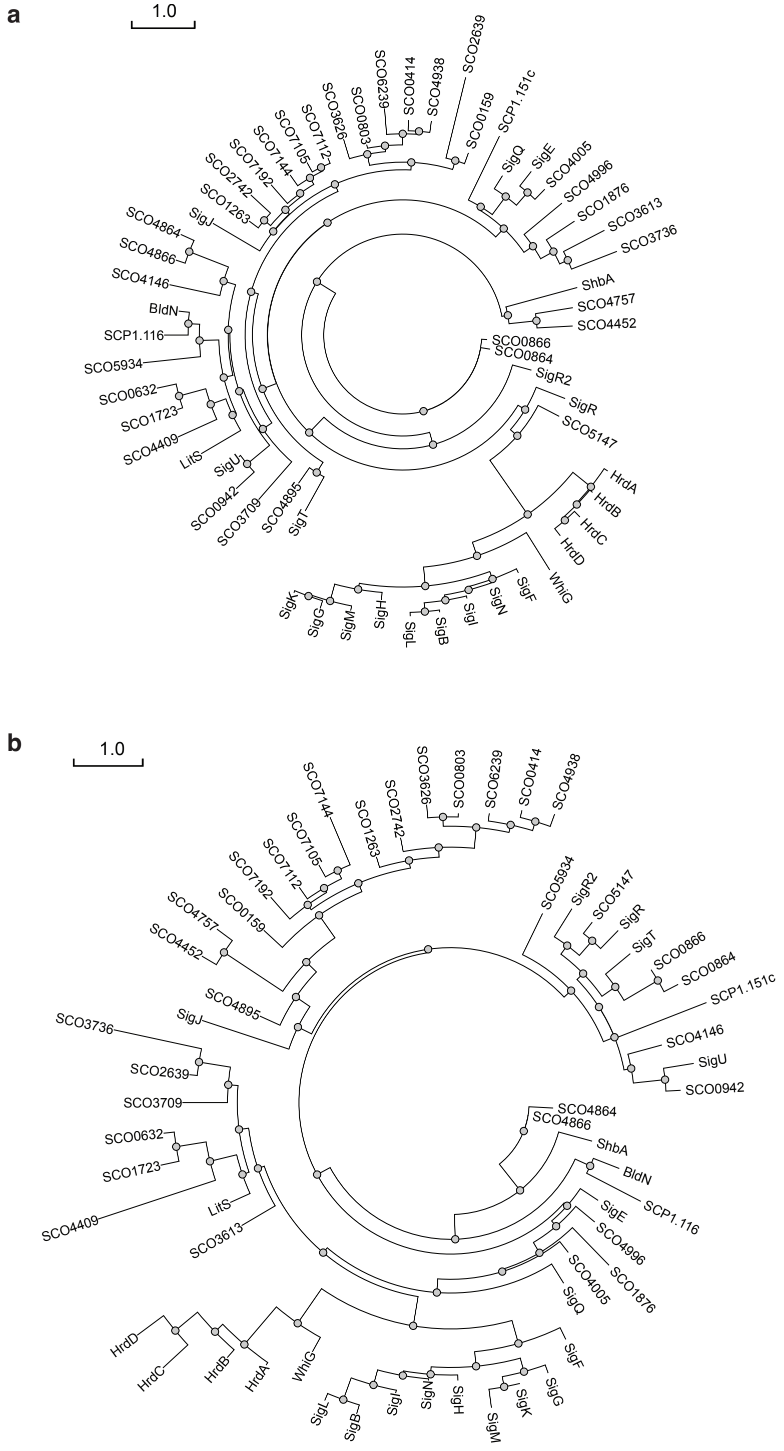

Supplementary Figure 4. Phylogenetic trees of (**a**) region 2 and (**b**) region 4 of all the sigma factors encoded in *S. coelicolor* A3(2). PhyML was used to estimate the phylogeny by maximum likelihood[1].

a

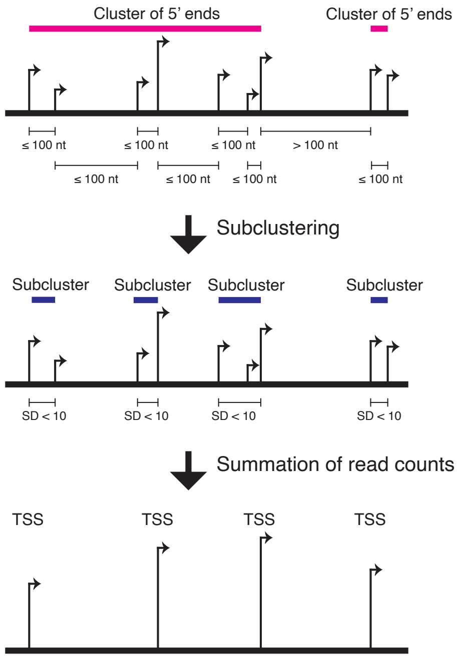

b

	No sigma factor			With a sigma factor		
	1	2	3	1	2	3
ERCC0013	13	20	5	11	13	22
ERCC0136	100	150	50	80	110	200
ERCC0138	80	100	20	60	90	110
ERCC0145	30	40	12	25	28	50

↓ Removing ERCC without sufficient read count(s)

	No sigma factor			With a sigma factor		
	1	2	3	1	2	3
ERCC0136	100	150	50	80	110	200
ERCC0138	80	100	20	60	90	110
ERCC0145	30	40	12	25	28	50

↓ Normalisation factor calculation

	No sigma factor			With a sigma factor		
	1	2	3	1	2	3
ERCC0136	0.5	0.75	0.25	0.4	0.55	1
ERCC0138	0.73	0.91	0.18	0.55	0.82	1
ERCC0145	0.6	0.8	0.24	0.5	0.56	1

↓ Mean nomalisation factor calculation

	No sigma factor			With a sigma factor		
	1	2	3	1	2	3
Normalisation factor	0.61	0.82	0.22	0.48	0.64	1

Supplementary Figure 5. Schemes of the TSS identification procedure. **a.** Clustering, subclustering and read count calculation of 5'-ends. Bent arrows indicate the 5'-ends of transcripts identified by 5'-end sequencing. **b.** Calculation of normalisation factors. The ERCC0013 had the read count smaller than 10 in the 3rd replicate of the "No sigma factor" sample. Therefore, ERCC0013 is excluded for the normalisation factor calculation in this example.

Supplementary Table 1. Previously characterised sigma factors.

Locus tag	Symbol	Organism[a]	Regulon[b]	Reference
SCO0194	LitS	*S. coelicolor*	*crt*	[2]
SCO0600	SigB	*S. coelicolor*	*sigB*, *catB*	[3]
SCO0895	HrdC	*S. coelicolor*	ND	[4]
SCO1276	SigJ	*S. coelicolor*	ND	[5]
SCO2465	HrdA	*S. coelicolor*	ND	[4]
SCO2954	SigU	*S. coelicolor*	ND	[6]
SCO3068	SigI	*S. coelicolor*	ND	[7]
SCO3202	HrdD	*S. coelicolor*	*actII-ORF4*, *redD*, *whiB*	[4,8,9]
SCO3323	BldN	*S. coelicolor*<br>*S. venezuelae*<br>*S. griseus*	*bldM*, *chp*, *rdl*, *rsbN*[c]	[10-12]
SCO3356	SigE	*S. coelicolor*	Cell envelope stress response	[13-15]
SCO3892	SigT	*S. coelicolor*	*clpP1P2*	[16]
SCO4005		*S. coelicolor*	ND	[17]
SCO4034	SigN	*S. coelicolor*	ND	[18,19]
SCO4035	SigF	*S. coelicolor*	*sspA*	[20,21]
SCO4452		*S. venezuelae*	ND	[22]
SCO4757		*S. venezuelae*	ND	[22]
SCO4769	ShbA	*S. griseus*	*hrdB*[d]	[23]
SCO4908	SigQ	*S. coelicolor*	ND	[24]
SCO5216	SigR	*S. coelicolor*	Thiol-oxidative stress response	[25-27]
SCO5243	SigH	*S. coelicolor*<br>*S. griseus*	*ssgB*, *gltB*, *prsH*/*rshA*, *sigJ*	[5,28-30]
SCO5621	WhiG	*S. coelicolor*<br>*S. venezuelae*	*whiH*, *whiI*[e]	[31,32]
SCO5820	HrdB	*S. avermitilis*<br>*S. coelicolor*<br>*S. griseus*	Housekeeping genes	[9,23,33-36]
SCO6520	SigK	*S. coelicolor*	ND	[37]
SCO7278	SigL	*S. coelicolor*	ND	[38]
SCO7314	SigM	*S. coelicolor*	ND	[38,39]
SCO7341	SigG	*S. coelicolor*	ND	[40]

[a]Organism in which the sigma factor was characterized.
[b]General category if more than 5 target genes/transcriptional units are known. ND: Not determined.
[c]Majority of target genes of BldN only confirmed in *Streptomyces venezuelae*.
[d]Target gene of ShbA only confirmed in *Streptomyces griseus*.
[e]Target genes of WhiG only confirmed in *S. venezuelae*.

Supplementary Table 2. Cloning and purification.

Locus tag	Primer[a]	Primer[b]	Cloned	Purified
SCO0159	5'linker-ATCCGCGTGGACAGCACCGCCACTG	3'linker-CCACTGGGCGATGAGCGGGAAGTTT	+	–
SCO0159_dC	5'linker-ATCCGCGTGGACAGCACCGCCACTG	3'linker-CGAGGTCGCAGCGGCGAGGAACTCTTC	+	–
SCO0194	5'linker-AACACGCGTACACGACCGACCACCAC	3'linker-GACACACGTGTCATGTGCGGCGCCC	+	–
SCO0414	5'linker-GGGACGGCTGGGACGAGTACCGACG	3'linker-GCCTACCCACGGCCCCAGCTTCTCG	+	–
SCO0414_dC	5'linker-GGGACGGCTGGGACGAGTACCGACG	3'linker-GGTCTGCCACGCCTCCTTGACCTGC	+	–
SCO0600	5'linker-ACGACGACGACCGCGCGAGCCACCG	3'linker-GGTGGTGCTGAGCATGCCTTCCCGC	+	–
SCO0632	5'linker-AAGGAAGCCGTGCACATCGGCAG	3'linker-CGCGCTCACCCCCAGGCAGTCACGG	+	–
SCO0803	5'linker-CGGGGACAGGGCGACATGCGGGGAG	3'linker-GGCCTCCAGCCCGGACACGTCCAAC	+	–
SCO0803_dC	5'linker-CGGGGACAGGGCGACATGCGGGGAG	3'linker-GGCGCGGCTGGCGGCGAGGAACGCC	+	–
SCO0864	5'linker-ACCCCTGCCCTGCCCACCGCGACCG	3'linker-GGCCGCGGCCCGCTCCACCGGGTGG	+	+
SCO0866	5'linker-ATCAGTCCAGCCCTGTCCGCCGCATG	3'linker-GGCCGAAGCCCTCTGAGCGGCGGGC	+	–
SCO0895	5'linker-GCGCCCACGGCACGCACACCGACGG	3'linker-GCTCGCCCAGTCCAGCAGGCGTTCC	+	–
SCO0942	5'linker-ACGGCCGGAATCACCCTCACGGACAC	3'linker-CCGCAGGTCGGCGGCGTATCCCGGT	+	–
SCO1263	5'linker-ACCACCGACACCGCGACCGACGTC	3'linker-GAGAACACCGGCCAGCGATACCAGT	+	–
SCO1263_dC	5'linker-ACCACCGACACCGCGACCGACGTC	3'linker-CTCGGCAGCGGCGGCGAGGAAGCGCTCG	+	–
SCO1276	5'linker-GCCGAACAAATGGAAAGTCGGCCGC	3'linker-TCGGGCGCAGCCTTCCAAGGCTTTC	+	–
SCO1564	5'linker-ACGCACGACCTGGTCACCTCCCTGC	3'linker-GCCCGCCGCAACCTCCGGCGTCAGC	+	–
SCO1723	5'linker-GCGGGCGACCGGGTGGCGGCGGACG	3'linker-TGCGACGCCGCCCAGGCACTCGCGC	–	–
SCO1876	5'linker-GGGACAGTCGTCGACGACGCCGCCG	3'linker-CCGGGCCCCTCCCGCCGTCTCACCG	+	–
SCO2465	5'linker-CGAGGCGGACAGCGGCGAGCGAGCC	3'linker-GTCCAGGTAGCCCCTCAGCTGGTCC	+	–
SCO2639	5'linker-ACGTACGCCTCGCGCCGAAAGGCGG	3'linker-CACCACCTCGTCCTCGTGCAGGCGG	+	–
SCO2742	5'linker-CACGAGACACCGCCGGAACCGAACG	3'linker-CCGGCTCAGGCTCGCCTCCTCACGC	+	+
SCO2742_dC	5'linker-CACGAGACACCGCCGGAACCGAACG	3'linker-CGTCTCGACCGCACGTTGGAAGGCC	+	+
SCO2954	5'linker-GGCGTGCGCAAGGATTCCGTCGTGG	3'linker-CGCCGTCACCCCCCGCTCCTCCAGA	+	–
SCO3068	5'linker-TCACCCCGGCTCGACGGATCGCGTAC	3'linker-CTCCTCGACCGTGAGCCCCTTGCGC	+	+
SCO3202	5'linker-GCAACCCGTGCCGTCGCCCGTCGTAAG	3'linker-GGCCGCCGCCTCGAAGCCGGTGTCG	+	–
SCO3323	5'linker-TACCCACACGTCGGGGTTGAC	3'linker-GCGGGCGTCGTCGGGGAGCAGC	+	–

SCO3323_dN	5′linker-ATGGAGCTGGTCGAGCGGGCC	3′linker-GCGGGCGTCGTCGGGGAGCAGC	+	–
SCO3356	5′linker-GGCGAGGTGCTCGAGTTCGAGGAG	3′linker-GGCCGCGCAACGCTCCCGCTCCTCA	+	–
SCO3450	5′linker-GAATGCGGGTCAGGCAGAGGGGGTTTC	3′linker-CATCACACCTCGCTTGGGCGCCAGT	+	–
SCO3613	5′linker-CCGGTGATCGCGCCGATGCCCGCAG	3′linker-TGCGGGAGCCTCCATGGCCACCCTG	+	–
SCO3626	5′linker-CCCGCACGATCCGACGACATGGCCC	3′linker-GCCTATGCCCGTGATGTCCAGTTCG	+	+
SCO3626_dC	5′linker-CCCGCACGATCCGACGACATGGCCC	3′linker-GCCGCGGGTGGCCGCCAGGTAGGCG	+	–
SCO3709	5′linker-CGACCACCAGACGAACCGCGCACCG	3′linker-GCCATGTCGCACCTCCGGCAGGCGA	+	–
SCO3736	5′linker-AAACGGTCCCGTGACAGGGCGGCGTC	3′linker-AGGCGTGTGGTCAAGGCTTCTCCTC	+	–
SCO3892	5′linker-GCGGGCGGCGCGAGTCATGACGGTG	3′linker-CGCTCGTCCACCTCCGCCCTTCACT	+	–
SCO4005	5′linker-AAGGCACGCCAGCCGGTGCTGCTGC	3′linker-CCGAACGAACCCCAGCTCAGGGTCC	+	–
SCO4034	5′linker-TCCGCAGAACAGGGCAGCTCGAAGG	3′linker-GTCGGAGATGAGACCCTCGCGCAGC	+	–
SCO4035	5′linker-CCGGCCAGTACTGCGCCTCAAGCAC	3′linker-TGCGTCGATCCGGTTCGCGGACCTC	+	+
SCO4146	5′linker-AGCGAAACGAGAAGCGACGGGGAGC	3′linker-TGCCAGCGCCTCCCGCAGCTGCCGG	+	–
SCO4409	5′linker-GCGAAGAGAGACGTGCCGCCGCGCTG	3′linker-TGCCGCACCCCCGTACCCGGGCGGA	+	–
SCO4452	5′linker-GACGAGGCCCTGGTCAGGAGCCTCAC	3′linker-CCGGCGCCCACGGCGACGGGAGTTG	+	–
SCO4452_dC	5′linker-GACGAGGCCCTGGTCAGGAGCCTCAC	3′linker-GGAGTAGCCCTCGTTGAAGACGAGG	+	–
SCO4757	5′linker-GAAGCACAGCCCGCCGCGGCCTTCG	3′linker-ACCACCGGTCGCCTCTTGCGCCCGG	+	–
SCO4757_dC	5′linker-GAAGCACAGCCCGCCGCGGCCTTCG	3′linker-GTAGCCCTCGTTGAAGATCAGGTAG	+	–
SCO4769	5′linker-CGTGACGACGATGCGCCCCCG	3′linker-CTGCTCCGCCAGCGCCCGCAG	+	+
SCO4864	5′linker-ACCTTGGGGGGCGCGCATCGGAGCG	3′linker-TGTCGACTCCCCCAGCGTCCTGGGA	+	–
SCO4866	5′linker-CTGGGGGACGACGCGGAGCTGACCG	3′linker-CATGTCCTTCTGCGTCCGCGCACGC	+	–
SCO4895	5′linker-ACCAACGGCACCGTGACGACGGAGC	3′linker-CTGGACCTCGTCGGCCTCCGCTTCG	+	+
SCO4895_dC	5′linker-ACCACCCGATCCGCGCCGGTCACCG	3′linker-GCCCTCGAAGGCCGCGACATACCGC	+	–
SCO4908	5′linker-ACGGTGGTTGACGCGAACACGGGGG	3′linker-GGCGACCAGTTCGCCGAAGGCGTCC	+	–
SCO4938	5′linker-ACCACCCGATCCGCGCCGGTCACCG	3′linker-CGCGGCCGGGGCCGTCGCCCAGGAG	+	–
SCO4938_dC	5′linker-AGGGACGGGGACGGGCCGGTGCGGG	3′linker-GGTCTCCCAGGCCCGCTTGAGATCG	+	–
SCO4996	5′linker-AGGCAGGGGCGGGCGGACGAGTACG	3′linker-GGGTCTGGCGTACTCCGAGAGGTCC	+	–
SCO5147	5′linker-AACGACACCGCTGCTGACCACAGCC	3′linker-CGCGCTCGTGCCCCCTCCTCCCAGT	+	–
SCO5216	5′linker-GGTCCGGTCACTGGGACCGAC	3′linker-TGACCCCGAGCCTTTCGCTTC	+	+

SCO5243	5’linker-AGGGACGGGGACGGGCCGGTGCGGG	3’linker-CTCCTCGACGAGCAGCTTCTCCCGC	+	+
SCO5243_dN	5’linker-CGGCACGACCCGCAGGACCGCAGCG	3’linker-CTCCTCGACGAGCAGCTTCTCCCGC	+	+
SCO5621	5’linker-CCCCAGCACACCTCCGGGTCCGACC	3’linker-GCGGCCGAAACCCGCAAGTTTGGCG	+	–
SCO5820	5’linker-TCGGCCAGCACATCCCGTAC	3’linker-GTCGAGGTAGTCGCGCAGCAC	+	+
SCO5820_dN	5’linker-CCCGAGGGTACCGAGAACGCGG	3’linker-GTCGAGGTAGTCGCGCAGCAC	+	+
SCO5934	5’linker-TCCGACCGGGAGCTGTGGGCGAGGG	3’linker-TCGTGCGACCTCCTTCTCGTTGACG	+	–
SCO6239	5’linker-CCGGAAGACACACCTGCGGAATCCG	3’linker-GTCGAGTGGCCAATTCGTCAGCTTC	+	+
SCO6239_dC	5’linker-CCGGAAGACACACCTGCGGAATCCG	3’linker-TCCGACAGTTGCGTCACGGAAAGCC	+	–
SCO6520	5’linker-CCGATCCACGCCAGCGTGAAGCACC	3’linker-CGCCTGCGGACCCGGATCCCGCAGG	+	+
SCO7099	5’linker-GGTGGAACCGGTGACACGACACCGC	3’linker-CACGTGCCCGACCGCTCCGTGTCCG	+	–
SCO7099_dC	5’linker-GGTGGAACCGGTGACACGACACCGC	3’linker-GAGCACGCAGGCCTGCCGGACGGCG	+	+
SCO7105	5’linker-GCAGGCGACCTGAGCGAGGCCGTTTC	3’linker-GACGCAGAGGGCCGCCACGGACCGG	+	–
SCO7105_dC	5’linker-GCAGGCGACCTGAGCGAGGCCGTTTC	3’linker-CTGCCGGGCGGCGGCCACGAACGTC	+	–
SCO7112	5’linker-CACGCTGGCGCCCCACCCGGCACCC	3’linker-GTCCCGCAGGGAAGCCACGTAGGGC	+	–
SCO7112_dC	5’linker-CACGCTGGCGCCCCACCCGGCACCC	3’linker-CGTCCGCGCCGCGGAGAGGAAGACC	+	–
SCO7144	5’linker-TCCGCGCCCAGCCCGGAGACCACCAC	3’linker-CGCGGCCTGACGGGCCGGGACGTGC	+	–
SCO7192	5’linker-GGCGCCAAGAGGGTTGGGCAACACG	3’linker-TGCGGCCTCGTAGTCCTCGGCCCCG	+	–
SCO7278	5’linker-CAGACCGCCGTGGTCCGTCCGCAAG	3’linker-GGCGCAGCCGAGTTCGCCCAGCATG	+	–
SCO7314	5’linker-CTCATAGAAACGCCCACCATCCGTC	3’linker-CCTGCTGTGTCCCCTGTGATCCTTG	+	–
SCO7341	5’linker-CGTGTCGCCGGCAGAACCCAGCCCC	3’linker-GCGGGCCTCCGCCAGGATCTCCTCA	+	–

[a] 5’linker: TTAAGAAGGAGATATACATATG

[b] 3’linker: GGATCTCAGTGGTGGTGGTGGTGGTGCTC

Supplementary Table 3. Direct target genes of SCO0864.

Target genes	Symbol	Annotation	Fold change[a]	Adjusted P value[a]
SCO4415		hypothetical protein	2.18	3.95×10^{-7}
SCO4693		hypothetical protein	2.08	3.5×10^{-15}
SCO4694		hypothetical protein	2.03	5.41×10^{-10}

[a]Fold changes and adjusted P values were determined by using the *DESeq2* package.

Supplementary Table 4. Direct target genes of SCO2742.

Target genes	Symbol	Annotation	Fold change[a]	Adjusted P value[a]
SCO7189		hypothetical protein	2.2	1.95×10^{-5}

[a]Fold changes and adjusted P values were determined by using the *DESeq2* package.

Supplementary Table 5. Direct target genes of SCO3068 (SigI).

Target genes	Symbol	Annotation	Fold change[a]	Adjusted P value[a]
SCO0960		hydrolase	2.45	3.39×10^{-24}
SCO1149		hypothetical protein	4.04	3.15×10^{-20}
SCO1221		transcription regulator AsnC	3.91	2.32×10^{-28}
SCO1276	*sigJ*	RNA polymerase ECF sigma factor	4.58	4.5×10^{-34}
SCO1531	*thrS*	threonyl-tRNA synthetase	2.04	3.19×10^{-11}
SCO1645		hypothetical protein	3.84	8.48×10^{-30}
SCO1646	*pup*	prokaryotic ubiquitin_like protein	2.21	0.00081
SCO1746	*sal*	serine protease	2.33	1.69×10^{-13}
SCO1793		hypothetical protein	4.37	1.2×10^{-42}
SCO2574		hypothetical protein	2.6	0.000352
SCO2870		hypothetical protein	3.17	6.04×10^{-12}
SCO2871		hypothetical protein	2.86	2.77×10^{-17}
SCO3424		regulator	2.16	1.41×10^{-5}
SCO3549	*bldG*	anti-sigma factor antagonist	2.16	1.51×10^{-5}
SCO3659		hypothetical protein	3.94	3.25×10^{-33}
SCO3660		hypothetical protein	2.07	1.43×10^{-13}
SCO3793		hypothetical protein	5.4	6.31×10^{-37}
SCO3933		regulatory protein	2.68	2.52×10^{-8}
SCO3987		hypothetical protein	2.15	0.00424
SCO4061		hypothetical protein	2.19	1.18×10^{-6}
SCO4337		integral membrane efflux protein	2.52	3.49×10^{-15}
SCO4421		TetR family transcriptional regulator	2.71	8.13×10^{-21}
SCO4514		hypothetical protein	2.17	1.62×10^{-6}
SCO4515		hypothetical protein	3.79	3.84×10^{-48}
SCO4563	*nuoB*	NADH dehydrogenase subunit B	2.7	1.14×10^{-8}
SCO4602	*nuoH2*	NADH dehydrogenase subunit NuoH2	2.04	2.68×10^{-7}
SCO4630		hypothetical protein	2.12	1.9×10^{-14}
SCO4648	*rplK*	50S ribosomal protein L11	2.08	2.87×10^{-5}
SCO4653	*rplL*	50S ribosomal protein L7/L12	2.29	2.23×10^{-5}
SCO5023		hypothetical protein	3.28	4.15×10^{-26}
SCO5085	*actII-4*	actinorhodin operon activator protein	3.08	1.11×10^{-24}
SCO5190	*wblC*	DNA-binding protein	2.55	1.16×10^{-6}
SCO5774	*gluD*	glutamate permease	2.14	1.0×10^{-9}
SCO5776	*gluB*	glutamate binding protein	2.24	5.93×10^{-13}
SCO5812	*rnhB*	ribonuclease HII	2.11	3.24×10^{-6}
SCO6269	*cpkPb*	2-oxoglutarate ferredoxin oxidoreductase subunit β	2.67	2.34×10^{-6}
SCO6633		transcriptional regulator	2.85	2.46×10^{-26}
SCO7199		hypothetical protein	2.13	0.00203
SCO7781		pseudogene, transposase remnant	2.58	5.33×10^{-8}
SCO7802			2.05	9.57×10^{-8}

[a]Fold changes and adjusted P values were determined by using the *DESeq2* package.

Supplementary Table 6. Direct target genes of SCO4035 (SigF).

Target genes	Symbol	Annotation	Fold change[a]	Adjusted P value[a]
SCO1276	*sigJ*	RNA polymerase ECF sigma factor	2.31	1.43×10^{-11}
SCO4187		hypothetical protein	2.69	4.92×10^{-19}
SCO4189		hypothetical protein	2.48	2.38×10^{-9}

[a]Fold changes and adjusted P values were determined by using the *DESeq2* package.

Supplementary Table 7. Direct target genes of SCO4769 (ShbA).

Target genes	Symbol	Annotation	Fold change[a]	Adjusted P value[a]
SCO4769	*shbA*	RNA polymerase sigma factor ShbA	9.4	5.88×10^{-99}
SCO5820	*hrdB*	RNA polymerase sigma factor HrdB	2.25	1.04×10^{-15}

[a]Fold changes and adjusted P values were determined by using the *DESeq2* package.

Supplementary Table 8. Direct target genes of SCO4895.

Target genes	Symbol	Annotation	Fold change[a]	Adjusted P value[a]
SCO0108		hypothetical protein	2.2	1.96×10^{-5}
SCO0874		hypothetical protein	2.19	1.26×10^{-5}
SCO0875		transcription regulator ArsR	3.5	7.55×10^{-5}
SCO1793		hypothetical protein	2.4	7.35×10^{-8}
SCO4512		hypothetical protein	2.5	3.08×10^{-7}
SCO4513		hypothetical protein	2.31	3.53×10^{-9}
SCO4514		hypothetical protein	2.27	4.33×10^{-7}
SCO4515		hypothetical protein	2.17	2.44×10^{-9}
SCO5646		solute binding lipoprotein	2.36	6.43×10^{-11}
SCO5718		hypothetical protein	2.1	9.03×10^{-9}
SCO5907		hypothetical protein	2.1	0.00423
SCO6404		hypothetical protein	2.32	0.000315
SCO7771		pseudogene, conserved hypothetical protein	2.04	9.66×10^{-6}
SCO7787		hypothetical protein	2.74	7.07×10^{-9}
SCO7788		hypothetical protein	2.7	0.00265

[a]Fold changes and adjusted P values were determined by using the *DESeq2* package.

Supplementary Table 9. Direct target genes of SCO5216 (SigR).

Target genes	Symbol	Annotation	Fold change[a]	Adjusted P value[a]
SCO0242		hypothetical protein	2.39	8.98×10^{-7}
SCO0562		hypothetical protein	2.55	1.8×10^{-18}
SCO0641		tellurium resistance protein	2.16	6.13×10^{-8}
SCO0680		transmembrane efflux protein	2.02	6.74×10^{-8}
SCO0875		transcription regulator ArsR	3.32	8.58×10^{-6}
SCO0877		transcriptional regulator	3.26	5.37×10^{-7}
SCO1089		hypothetical protein	2.19	1.92×10^{-12}
SCO1200		regulatory protein	2.02	9.28×10^{-5}
SCO1421	*rbpA*	RNA polymerase-binding protein RbpA	3.29	1.47×10^{-26}
SCO1429	*chiD*	chitinase	2.1	1.98×10^{-10}
SCO1598	*rplT*	50S ribosomal protein L20	3.02	1.37×10^{-8}
SCO1793		hypothetical protein	3.67	3.81×10^{-34}
SCO1814	*fabI/inhA*	enoyl-ACP reductase	2	1.7×10^{-5}
SCO1839		transcriptional regulator	2.23	0.00087
SCO1864	*ectA*	L-2,4-diaminobutyrate acetyltransferase	2.26	2.5×10^{-10}
SCO1995		hypothetical protein	2.41	4.85×10^{-6}
SCO1997	*asp1*	actinobacteria-specific protein	3.41	2.78×10^{-32}
SCO2303		hypothetical protein	2.77	2.42×10^{-8}
SCO2493		hypothetical protein	2.33	1.95×10^{-12}
SCO2610	*mreC*	rod shape-determining protein MreC	2.62	3.17×10^{-20}
SCO2676		hypothetical protein	2.09	0.000124
SCO3043		hypothetical protein	2.11	1.35×10^{-11}

SCO3202	*hrdD*	RNA polymerase principal sigma factor	4.28	4.45×10^{-50}
SCO3270		hypothetical protein	2.02	1.42×10^{-7}
SCO3429	*rpmB*	50S ribosomal protein L28	2.31	1.91×10^{-5}
SCO3506		LacI family transcriptional regulator	2.05	1.95×10^{-9}
SCO3536			2.91	7.46×10^{-8}
SCO3596	*vanX*	D-alanine:D-alanine dipeptidase	2.21	1.67×10^{-8}
SCO3793		hypothetical protein	3.25	2.66×10^{-17}
SCO3810		GntR family transcriptional regulator	3.19	8.24×10^{-15}
SCO3890	*trxB*	thioredoxin reductase	2.67	5.43×10^{-25}
SCO3954		short chain dehydrogenase	2.06	9.99×10^{-12}
SCO4061		hypothetical protein	2.15	1.51×10^{-7}
SCO4198		DNA-binding protein	2.25	6.17×10^{-6}
SCO4212		hypothetical protein	2.87	3.96×10^{-19}
SCO4213		hypothetical protein	2.15	2.03×10^{-14}
SCO4465		hypothetical protein	2.65	3.44×10^{-13}
SCO4498		proton transport protein	2.16	3.59×10^{-11}
SCO4514		hypothetical protein	2.38	1.98×10^{-10}
SCO4599	*nuoA2*	NADH dehydrogenase subunit NuoA2	2.06	1.07×10^{-5}
SCO4835		hypothetical protein	2.37	4.46×10^{-8}
SCO4997		hypothetical protein	2.16	0.000303
SCO5163		hypothetical protein	12.25	5.46×10^{-120}
SCO5178	*moeB*	adenylyltransferase/sulfurtransferase MoeZ	4.19	6.79×10^{-56}
SCO5240	*wblE*	hypothetical protein	2.09	1.19×10^{-5}
SCO5287		MarR family transcriptional regulator	2.08	1.48×10^{-5}
SCO5359	*rpmE1*	50S ribosomal protein L31	2.97	9.32×10^{-10}
SCO5365		transferase	2.39	1.27×10^{-17}
SCO5619		hypothetical protein	2.89	9.48×10^{-18}
SCO5634		hypothetical protein (pseudogene)	2.22	7.69×10^{-7}
SCO5639		hypothetical protein	2.22	1.24×10^{-8}
SCO5796	*hflX*	hypothetical protein	2.77	5.15×10^{-23}
SCO5837		zinc protease	2.27	2.81×10^{-14}
SCO5900		hypothetical protein	2.85	2.53×10^{-27}
SCO6246	*allR*	transcriptional regulator for glyoxylate bypass	2.59	1.42×10^{-11}
SCO6247	*allB*	allantoinase	2.02	1.3×10^{-6}
SCO6269	*cpkPb*	2-oxoglutarate ferredoxin oxidoreductase subunit β	3.43	7.23×10^{-12}
SCO6308		hydrolase	2.73	1.59×10^{-18}
SCO6404		hypothetical protein	4.74	2.81×10^{-22}
SCO6405		DNA recombinase	2.62	1.16×10^{-28}
SCO6480		hypothetical protein	2.23	2.0×10^{-5}
SCO6531		ATP/GTP binding protein	2.22	7.41×10^{-7}
SCO6650		hypothetical protein	2.05	0.000616
SCO6863		hypothetical protein	2.11	1.49×10^{-8}
SCO7201		transcriptional regulator	2.37	3.67×10^{-7}
SCO7420	*cvnC10*	hypothetical protein	2.9	4.61×10^{-7}
SCO7666		zinc-binding oxidoreductase (fragment)	2.3	6.79×10^{-10}
SCO7722		hypothetical protein	2.89	1.13×10^{-11}
SCO7767		DNA-binding protein	2.55	6.84×10^{-17}
SCO7787		hypothetical protein	2.72	8.93×10^{-12}
SCO7788		hypothetical protein	3.47	8.29×10^{-8}

[a]Fold changes and adjusted P values were determined by using the *DESeq2* package.

Supplementary Table 10. Direct target genes of SCO5243 (SigH).

Target genes	Symbol	Annotation	Fold change[a]	Adjusted P value[a]
SCO1149		hypothetical protein	2.76	2.91×10^{-9}
SCO1276	*sigJ*	RNA polymerase ECF sigma factor	5.49	1.78×10^{-62}
SCO1644	*prcB*	20S proteasome subunit β	2.01	1.23×10^{-5}

SCO1645		hypothetical protein	2.87	9.76×10^{-22}
SCO1793		hypothetical protein	3.61	3.77×10^{-31}
SCO1863		hypothetical protein	6.05	4.91×10^{-42}
SCO2352		hypothetical protein	2.42	0.00463
SCO2627		ribose-5-phosphate isomerase B	2.39	9.68×10^{-8}
SCO2870		hypothetical protein	2.26	8.73×10^{-6}
SCO2871		hypothetical protein	2.08	1.59×10^{-8}
SCO3484		sugar-binding protein	2.5	2.15×10^{-16}
SCO3793		hypothetical protein	3.52	1.69×10^{-17}
SCO3955		hypothetical protein	2.16	4.49×10^{-8}
SCO4187		hypothetical protein	5.1	5.82×10^{-64}
SCO4189		hypothetical protein	5.6	4.12×10^{-36}
SCO4346		hypothetical protein	2.98	5.48×10^{-50}
SCO4512		hypothetical protein	2.56	1.59×10^{-8}
SCO4513		hypothetical protein	2.34	1.14×10^{-10}
SCO4514		hypothetical protein	2.28	2.63×10^{-9}
SCO4515		hypothetical protein	2.78	1.68×10^{-31}
SCO4516		hypothetical protein	2.29	5.21×10^{-9}
SCO5023		hypothetical protein	2.11	2.68×10^{-14}
SCO5190	*wblC*	DNA-binding protein	2.04	0.000348
SCO5243	*sigH*	RNA polymerase sigma factor	571.49	0
SCO5646		solute binding lipoprotein	2.2	1.01×10^{-13}
SCO6317		hypothetical protein	2.57	7.19×10^{-9}
SCO7189		hypothetical protein	2.63	1.88×10^{-8}

[a]Fold changes and adjusted P values were determined by using the *DESeq2* package.

Supplementary Table 11. Direct target genes of SCO5820 (HrdB).

Target genes	Symbol	Annotation	Fold change[a]	Adjusted P value[a]
SCO7031	*bxlA*	β-D-xylosidase	2.98	0.000454

[a]Fold changes and adjusted P values were determined by using the *DESeq2* package.

Supplementary Table 12. Direct target genes of SCO6239.

Target genes	Symbol	Annotation	Fold change[a]	Adjusted P value[a]
SCO4693		hypothetical protein	2.04	3.91×10^{-10}
SCO6261		hypothetical protein	2.29	0.00532

[a]Fold changes and adjusted P values were determined by using the *DESeq2* package.

Supplementary Table 13. Direct target genes of SCO6520 (SigK).

Target genes	Symbol	Annotation	Fold change[a]	Adjusted P value[a]
SCO0904		hypothetical protein	2.29	0.00891
SCO5190	*wblC*	DNA-binding protein	2.15	4.84×10^{-5}

[a]Fold changes and adjusted P values were determined by using the *DESeq2* package.

Supplementary Table 14. Direct target genes of SCO2742 ΔC.

Target genes	Symbol	Annotation	Fold change[a]	Adjusted P value[a]
SCO0875		transcription regulator ArsR	2.52	0.0037
SCO1793		hypothetical protein	2.08	1.35×10^{-10}
SCO2562	*lepA*	GTP-binding protein LepA	2.07	1.62×10^{-18}
SCO3890	*trxB*	thioredoxin reductase	2.27	3.81×10^{-17}
SCO3955		hypothetical protein	2.28	6.32×10^{-12}
SCO5619		hypothetical protein	2.2	6.37×10^{-10}
SCO7788		hypothetical protein	2.15	0.00789

[a]Fold changes and adjusted P values were determined by using the *DESeq2* package.

Supplementary Table 15. Direct target genes of SCO5243 (SigH) ΔN.

Target genes	Symbol	Annotation	Fold change[a]	Adjusted P value[a]
SCO0858		hypothetical protein	2.59	2.57×10^{-17}
SCO0875		transcription regulator ArsR	3.11	0.000156
SCO0960		hydrolase	2.07	1.94×10^{-17}
SCO1221		transcription regulator AsnC	2.57	1.78×10^{-15}
SCO1276	*sigJ*	RNA polymerase ECF sigma factor	7.05	3.72×10^{-94}
SCO1277		hypothetical protein	3.55	1.67×10^{-16}
SCO1278		ATP/GTP-binding protein	2.3	2.21×10^{-15}
SCO1279		hypothetical protein	2.23	9.03×10^{-7}
SCO1645		hypothetical protein	3.05	4.62×10^{-22}
SCO1793		hypothetical protein	3.48	4.81×10^{-26}
SCO1863		hypothetical protein	6.34	2.01×10^{-12}
SCO2563	*rpsT*	30S ribosomal protein S20	2.53	9.22×10^{-9}
SCO2627		ribose-5-phosphate isomerase B	2.54	6.13×10^{-11}
SCO2870		hypothetical protein	2.19	3.62×10^{-5}
SCO2871		hypothetical protein	2.51	9.76×10^{-17}
SCO3557		septum site determining protein	2.37	1.95×10^{-11}
SCO3793		hypothetical protein	3.09	4.05×10^{-15}
SCO3889	*trxA*	thioredoxin	2.09	1.03×10^{-8}
SCO3930		hypothetical protein	2.19	1.43×10^{-10}
SCO4187		hypothetical protein	5.7	8.01×10^{-73}
SCO4189		hypothetical protein	8.12	1.05×10^{-71}
SCO4337		integral membrane efflux protein	2.59	2.57×10^{-17}
SCO4398		hypothetical protein	2.17	4.05×10^{-5}
SCO4512		hypothetical protein	2.49	6.17×10^{-10}
SCO4513		hypothetical protein	2.14	3.21×10^{-9}
SCO4514		hypothetical protein	2.16	2.37×10^{-7}
SCO4515		hypothetical protein	2.82	1.6×10^{-31}
SCO4516		hypothetical protein	2.04	5.03×10^{-6}
SCO5023		hypothetical protein	2.49	3.25×10^{-22}
SCO5190	*wblC*	DNA-binding protein	2.13	0.000264
SCO6378		hypothetical protein	2.05	0.000166
SCO6633		transcriptional regulator	2.1	2.68×10^{-13}
SCO7189		hypothetical protein	2.07	0.000433

[a]Fold changes and adjusted P values were determined by using the *DESeq2* package.

Supplementary Table 16. Direct target genes of SCO5820 (HrdB) ΔN.

Target genes	Symbol	Annotation	Fold change[a]	Adjusted P value[a]
SCO1428	*acd*	acyl-CoA dehydrogenase	2.86	7.56×10^{-26}
SCO1699		transcriptional regulator	2.18	7.98×10^{-6}
SCO2252		hypothetical protein	4.53	1.21×10^{-13}
SCO3092		oxidoreductase	2.06	7.36×10^{-31}
SCO3464		hypothetical protein	2.45	4.43×10^{-16}
SCO3793		hypothetical protein	2.16	4.05×10^{-9}
SCO4449		hydrolase	3.66	8.58×10^{-37}
SCO4654	*rpoB*	DNA-directed RNA polymerase subunit β	2.65	2.44×10^{-44}
SCO5366	*atpI*	ATP synthase I	2.06	1.84×10^{-8}
SCO5519		hypothetical protein	2.73	2.65×10^{-24}
SCO7420	*cvnC10*	hypothetical protein	2.28	0.00014
SCO7788		hypothetical protein	2.57	2.69×10^{-5}
SCO7789		hypothetical protein	2.46	1.2×10^{-5}
SCO7790		oxidoreductase	2.55	4.39×10^{-9}

[a]Fold changes and adjusted P values were determined by using the *DESeq2* package.

Supplementary Table 17. Primers used for quantitative RT-PCR.

Name	Sequence (5' to 3')	Gene/transcript
hrdB_F	ATTGAGCGGGGAAAGGCTG	*hrdB*
hrdB_R	TCGAGGATCTGGTTGAGGCT	*hrdB*
trxB_F	GAGCTCATGGACAACATGCG	*trxB*
trxB_R	CGGTGACGGTCTTGATCTCG	*trxB*
ERCC0095_F	CTTGCCTGCTGCATGTTGTG	ERCC0095
ERCC0095_R	GAGCGATAGCGGTTAAGCCA	ERCC0095
ERCC0096_F	AAACCGACGCAGTTTTGCTC	ERCC0096
ERCC0096_R	ACTGGACACTGCATCGGAAG	ERCC0096
ERCC0136_F	ACAACTGCTTAAAGCGTGCG	ERCC0136
ERCC0136_R	TCTTTGCGAAGTCCGAGTCC	ERCC0136
ERCC0145_F	GCCATATCGGCTCGCAAATC	ERCC0145
ERCC0145_R	GAATGCCGAAACCTCCTCCA	ERCC0145

REFERENCES

1 Guindon, S. *et al.* New algorithms and methods to estimate maximum-likelihood phylogenies: assessing the performance of PhyML 3.0. *Syst Biol* **59**, 307-321, doi:10.1093/sysbio/syq010 (2010).

2 Takano, H., Obitsu, S., Beppu, T. & Ueda, K. Light-induced carotenogenesis in *Streptomyces coelicolor* A3(2): identification of an extracytoplasmic function sigma factor that directs photodependent transcription of the carotenoid biosynthesis gene cluster. *J Bacteriol* **187**, 1825-1832, doi:10.1128/JB.187.5.1825-1832.2005 (2005).

3 Cho, Y. H., Lee, E. J., Ahn, B. E. & Roe, J. H. SigB, an RNA polymerase sigma factor required for osmoprotection and proper differentiation of *Streptomyces coelicolor*. *Mol Microbiol* **42**, 205-214, doi:10.1046/j.1365-2958.2001.02622.x (2001).

4 Buttner, M. J. & Lewis, C. G. Construction and characterization of *Streptomyces coelicolor* A3(2) mutants that are multiply deficient in the nonessential *hrd*-encoded RNA polymerase sigma factors. *J Bacteriol* **174**, 5165-5167, doi:10.1128/jb.174.15.5165-5167.1992 (1992).

5 Mazurakova, V., Sevcikova, B., Rezuchova, B. & Kormanec, J. Cascade of sigma factors in streptomycetes: identification of a new extracytoplasmic function sigma factor σ^J that is under the control of the stress-response sigma factor σ^H in *Streptomyces coelicolor* A3(2). *Arch Microbiol* **186**, 435-446, doi:10.1007/s00203-006-0158-9 (2006).

6 Gordon, N. D. *et al.* Secreted-protein response to σ^U activity in *Streptomyces coelicolor*. *J Bacteriol* **190**, 894-904, doi:10.1128/JB.01759-07 (2008).

7 Homerova, D., Sevcikova, B., Rezuchova, B. & Kormanec, J. Regulation of an alternative sigma factor σ^I by a partner switching mechanism with an anti-sigma factor PrsI and an anti-anti-sigma factor ArsI in *Streptomyces coelicolor* A3(2). *Gene* **492**, 71-80, doi:10.1016/j.gene.2011.11.011 (2012).

8 Fujii, T., Gramajo, H. C., Takano, E. & Bibb, M. J. *redD* and *act*II-ORF4, pathway-specific regulatory genes for antibiotic production in *Streptomyces coelicolor* A3(2), are transcribed *in vitro* by an RNA polymerase holoenzyme containing σ^{hrdD}. *J Bacteriol* **178**, 3402-3405, doi:10.1128/jb.178.11.3402-3405.1996 (1996).

9 Kang, J. G., Hahn, M. Y., Ishihama, A. & Roe, J. H. Identification of sigma factors for growth phase-related promoter selectivity of RNA polymerases from *Streptomyces coelicolor* A3(2). *Nucleic Acids Res* **25**, 2566-2573, doi:10.1093/nar/25.13.2566 (1997).

10 Bibb, M. J., Molle, V. & Buttner, M. J. σ^{BldN}, an extracytoplasmic function RNA polymerase sigma factor required for aerial mycelium formation in *Streptomyces coelicolor* A3(2). *J Bacteriol* **182**, 4606-4616, doi:10.1128/jb.182.16.4606-4616.2000 (2000).

11 Bibb, M. J., Domonkos, A., Chandra, G. & Buttner, M. J. Expression of the chaplin and rodlin hydrophobic sheath proteins in *Streptomyces venezuelae* is controlled by σ^{BldN} and a cognate anti-sigma factor, RsbN. *Mol Microbiol* **84**, 1033-1049, doi:10.1111/j.1365-2958.2012.08070.x (2012).

12 Yamazaki, H., Ohnishi, Y. & Horinouchi, S. An A-factor-dependent extracytoplasmic function sigma factor (σ^{AdsA}) that is essential for morphological development in *Streptomyces griseus*. *J Bacteriol* **182**, 4596-4605, doi:10.1128/jb.182.16.4596-4605.2000 (2000).

13 Tran, N. T. *et al.* Defining the regulon of genes controlled by σ^E, a key regulator of the cell envelope stress response in *Streptomyces coelicolor*. *Mol Microbiol* **112**, 461-481, doi:10.1111/mmi.14250 (2019).

14 Hong, H. J., Paget, M. S. & Buttner, M. J. A signal transduction system in *Streptomyces coelicolor* that activates the expression of a putative cell wall glycan operon in response to vancomycin and other cell wall-specific antibiotics. *Mol Microbiol* **44**, 1199-1211, doi:10.1046/j.1365-2958.2002.02960.x (2002).
15 Paget, M. S., Chamberlin, L., Atrih, A., Foster, S. J. & Buttner, M. J. Evidence that the extracytoplasmic function sigma factor σ^E is required for normal cell wall structure in *Streptomyces coelicolor* A3(2). *J Bacteriol* **181**, 204-211 (1999).
16 Mao, X. M. *et al.* Dual positive feedback regulation of protein degradation of an extra-cytoplasmic function sigma factor for cell differentiation in *Streptomyces coelicolor*. *J Biol Chem* **288**, 31217-31228, doi:10.1074/jbc.M113.491498 (2013).
17 Hesketh, A., Chen, W. J., Ryding, J., Chang, S. & Bibb, M. The global role of ppGpp synthesis in morphological differentiation and antibiotic production in *Streptomyces coelicolor* A3(2). *Genome Biol* **8**, R161, doi:10.1186/gb-2007-8-8-r161 (2007).
18 Dalton, K. A., Thibessard, A., Hunter, J. I. & Kelemen, G. H. A novel compartment, the 'subapical stem' of the aerial hyphae, is the location of a *sigN*-dependent, developmentally distinct transcription in *Streptomyces coelicolor*. *Mol Microbiol* **64**, 719-737, doi:10.1111/j.1365-2958.2007.05684.x (2007).
19 Wang, C. *et al.* SigN is responsible for differentiation and stress responses based on comparative proteomic analyses of *Streptomyces coelicolor* wild-type and *sigN* deletion strains. *Microbiol Res* **165**, 221-231, doi:10.1016/j.micres.2009.05.003 (2010).
20 Tzanis, A. *et al.* A sporulation-specific, *sigF*-dependent protein, SspA, affects septum positioning in *Streptomyces coelicolor*. *Mol Microbiol* **91**, 363-380, doi:10.1111/mmi.12466 (2014).
21 Kelemen, G. H. *et al.* Developmental regulation of transcription of *whiE*, a locus specifying the polyketide spore pigment in *Streptomyces coelicolor* A3 (2). *J Bacteriol* **180**, 2515-2521, doi:10.1128/JB.180.9.2515-2521.1998 (1998).
22 Liu, Q., Pinto, D. & Mascher, T. Characterization of the widely distributed novel ECF42 group of extracytoplasmic function σ factors in *Streptomyces venezuelae*. *J Bacteriol* **200**, doi:10.1128/JB.00437-18 (2018).
23 Otani, H., Higo, A., Nanamiya, H., Horinouchi, S. & Ohnishi, Y. An alternative sigma factor governs the principal sigma factor in *Streptomyces griseus*. *Mol Microbiol* **87**, 1223-1236, doi:10.1111/mmi.12160 (2013).
24 Shu, D. *et al.* *afsQ1-Q2-sigQ* is a pleiotropic but conditionally required signal transduction system for both secondary metabolism and morphological development in *Streptomyces coelicolor*. *Appl Microbiol Biotechnol* **81**, 1149-1160, doi:10.1007/s00253-008-1738-1 (2009).
25 Kim, M. S. *et al.* Conservation of thiol-oxidative stress responses regulated by SigR orthologues in actinomycetes. *Mol Microbiol* **85**, 326-344, doi:10.1111/j.1365-2958.2012.08115.x (2012).
26 Paget, M. S., Kang, J. G., Roe, J. H. & Buttner, M. J. σ^R, an RNA polymerase sigma factor that modulates expression of the thioredoxin system in response to oxidative stress in *Streptomyces coelicolor* A3(2). *EMBO J* **17**, 5776-5782, doi:10.1093/emboj/17.19.5776 (1998).
27 Paget, M. S., Molle, V., Cohen, G., Aharonowitz, Y. & Buttner, M. J. Defining the disulphide stress response in *Streptomyces coelicolor* A3(2): identification of the σ^R regulon. *Mol Microbiol* **42**, 1007-1020, doi:10.1046/j.1365-2958.2001.02675.x (2001).
28 Kormanec, J. & Sevcikova, B. The stress-response sigma factor σ^H controls the expression of *ssgB*, a homologue of the sporulation-specific cell division gene *ssgA*, in

Streptomyces coelicolor A3(2). *Mol Genet Genomics* **267**, 536-543, doi:10.1007/s00438-002-0687-0 (2002).

29 Kormanec, J. & Sevcikova, B. Stress-response sigma factor σ^H directs expression of the *gltB* gene encoding glutamate synthase in *Streptomyces coelicolor* A3(2). *Biochim Biophys Acta* **1577**, 149-154, doi:10.1016/s0167-4781(02)00409-8 (2002).

30 Takano, H., Hosono, K., Beppu, T. & Ueda, K. Involvement of σ^H and related sigma factors in glucose-dependent initiation of morphological and physiological development of *Streptomyces griseus*. *Gene* **320**, 127-135, doi:10.1016/s0378-1119(03)00818-7 (2003).

31 Gallagher, K. A. *et al.* c-di-GMP arms an anti-σ to control progression of multicellular differentiation in *Streptomyces*. *Mol Cell* **77**, 586-599 e586, doi:10.1016/j.molcel.2019.11.006 (2020).

32 Ryding, N. J. *et al.* A developmentally regulated gene encoding a repressor-like protein is essential for sporulation in *Streptomyces coelicolor* A3(2). *Mol Microbiol* **29**, 343-357, doi:10.1046/j.1365-2958.1998.00939.x (1998).

33 Buttner, M. J., Smith, A. M. & Bibb, M. J. At least three different RNA polymerase holoenzymes direct transcription of the agarase gene (*dagA*) of *Streptomyces coelicolor* A3(2). *Cell* **52**, 599-607, doi:10.1016/0092-8674(88)90472-2 (1988).

34 Smidova, K. *et al.* DNA mapping and kinetic modeling of the HrdB regulon in *Streptomyces coelicolor*. *Nucleic Acids Res* **47**, 621-633, doi:10.1093/nar/gky1018 (2019).

35 van Wezel, G. P., Takano, E., Vijgenboom, E., Bosch, L. & Bibb, M. J. The *tuf3* gene of *Streptomyces coelicolor* A3(2) encodes an inessential elongation factor Tu that is apparently subject to positive stringent control. *Microbiology* **141 (Pt 10)**, 2519-2528, doi:10.1099/13500872-141-10-2519 (1995).

36 Zhuo, Y. *et al.* Reverse biological engineering of *hrdB* to enhance the production of avermectins in an industrial strain of *Streptomyces avermitilis*. *Proc Natl Acad Sci U S A* **107**, 11250-11254, doi:10.1073/pnas.1006085107 (2010).

37 Mao, X. M., Zhou, Z., Hou, X. P., Guan, W. J. & Li, Y. Q. Reciprocal regulation between SigK and differentiation programs in *Streptomyces coelicolor*. *J Bacteriol* **191**, 6473-6481, doi:10.1128/JB.00875-09 (2009).

38 Lee, E. J. *et al.* A master regulator sigmaB governs osmotic and oxidative response as well as differentiation via a network of sigma factors in *Streptomyces coelicolor*. *Mol Microbiol* **57**, 1252-1264, doi:10.1111/j.1365-2958.2005.04761.x (2005).

39 Gaskell, A. A., Crack, J. C., Kelemen, G. H., Hutchings, M. I. & Le Brun, N. E. RsmA is an anti-sigma factor that modulates its activity through a [2Fe-2S] cluster cofactor. *J Biol Chem* **282**, 31812-31820, doi:10.1074/jbc.M705160200 (2007).

40 Kormanec, J., Homerova, D., Barak, I. & Sevcikova, B. A new gene, *sigG*, encoding a putative alternative sigma factor of *Streptomyces coelicolor* A3(2). *FEMS Microbiol Lett* **172**, 153-158, doi:10.1111/j.1574-6968.1999.tb13463.x (1999).
