# Supplementary material for: RIViT-seq enables systematic identification of regulons of transcriptional machineries
Source: Nat Commun. 2022 Jun 17;13:3502. doi: 10.1038/s41467-022-31191-w (PMC9205884; doi:10.1038/s41467-022-31191-w)
Supplement: Supplementary file 3 — Reporting Summary [file 41467_2022_31191_MOESM3_ESM.pdf]

nature portfolio

Corresponding author(s): Hiroshi Otani, Nigel J. Mouncey

Last updated by author(s): Apr 25, 2022

Reporting Summary

Nature Portfolio wishes to improve the reproducibility of the work that we publish. This form provides structure for consistency and transparency in reporting. For further information on Nature Portfolio policies, see our Editorial Policies and the Editorial Policy Checklist.

Statistics

For all statistical analyses, confirm that the following items are present in the figure legend, table legend, main text, or Methods section.

n/a	Confirmed	
☐	☒	The exact sample size (*n*) for each experimental group/condition, given as a discrete number and unit of measurement
☐	☒	A statement on whether measurements were taken from distinct samples or whether the same sample was measured repeatedly
☐	☒	The statistical test(s) used AND whether they are one- or two-sided<br>*Only common tests should be described solely by name; describe more complex techniques in the Methods section.*
☒	☐	A description of all covariates tested
☒	☐	A description of any assumptions or corrections, such as tests of normality and adjustment for multiple comparisons
☐	☒	A full description of the statistical parameters including central tendency (e.g. means) or other basic estimates (e.g. regression coefficient) AND variation (e.g. standard deviation) or associated estimates of uncertainty (e.g. confidence intervals)
☐	☒	For null hypothesis testing, the test statistic (e.g. *F*, *t*, *r*) with confidence intervals, effect sizes, degrees of freedom and *P* value noted<br>*Give P values as exact values whenever suitable.*
☒	☐	For Bayesian analysis, information on the choice of priors and Markov chain Monte Carlo settings
☒	☐	For hierarchical and complex designs, identification of the appropriate level for tests and full reporting of outcomes
☒	☐	Estimates of effect sizes (e.g. Cohen's *d*, Pearson's *r*), indicating how they were calculated

Our web collection on statistics for biologists contains articles on many of the points above.

Software and code

Policy information about availability of computer code

Data collection	No softwares were used for data collection.
Data analysis	For whole transcriptomics, the following softwares were used.<br>Raw reads were scanned from 3’ to 5’ and those with a quality score value below 20 were trimmed and reads consisting of fewer than 35 nucleotides were discarded using BBDuk v38.79. Trimmed reads were aligned to the S. coelicolor A3(2) chromosome and ERCC92 sequences using HISAT2 version 2.1.0 with the “no-spliced-alignment” option and the “maxins” option of 1,000. The number of fragments overlapping each gene was counted using featureCounts v2.0.0. Fragment counts were normalised by ERCC fragment counts using the R package RUVSeq v1.20.0 and differential expression was analysed using the R package DESeq2 v1.26.0.<br><br>For 5'-end sequencing, the following softwares were used.<br>Adaptor sequences present at the 3’-end of reads were trimmed using BBDuk v38.79. Raw reads were scanned from 3’ to 5’ and those with a quality score value below 20 were trimmed and reads consisting of fewer than 35 nucleotides were discarded using BBDuk (sourceforge.net/projects/bbmap/). Trimmed reads were aligned to the S. coelicolor A3(2) chromosome and ERCC92 sequences using HISAT2 version 2.1.0 with the “no-spliced-alignment” option and the “maxins” option of 1,000. The number of 5’-end of forward reads that aligned each genomic position was counted using Samtools v1.10.<br><br>MEME v5.3.3 was used to find consensus motifs. PhyML 3.3 was used to estimate the phylogeny by maximum likelihood. The hpc_hmmsearch algorithm and the Pfam database 33.1 were used for protein domain search.

For manuscripts utilizing custom algorithms or software that are central to the research but not yet described in published literature, software must be made available to editors and reviewers. We strongly encourage code deposition in a community repository (e.g. GitHub). See the Nature Portfolio guidelines for submitting code & software for further information.

Data

Policy information about availability of data

All manuscripts must include a data availability statement. This statement should provide the following information, where applicable:

- Accession codes, unique identifiers, or web links for publicly available datasets
- A description of any restrictions on data availability
- For clinical datasets or third party data, please ensure that the statement adheres to our policy

All sequence files and processed count data files are available at Gene Expression Omnibus (GEO) under accession number GSE184392 (whole transcriptome) and GSE184393 (5'-end sequencing).

Field-specific reporting

Please select the one below that is the best fit for your research. If you are not sure, read the appropriate sections before making your selection.

☒ Life sciences ☐ Behavioural & social sciences ☐ Ecological, evolutionary & environmental sciences

For a reference copy of the document with all sections, see nature.com/documents/nr-reporting-summary-flat.pdf

Life sciences study design

All studies must disclose on these points even when the disclosure is negative.

Sample size	No sample size calculation was performed. The DESeq2 package used for statistical analysis whole transcriptomics is able to estimate dispersions with at least 2 replicates. We used 4 replicates, twice as many as the number of replicates minimally required.
Data exclusions	No data were excluded.
Replication	All attempts at replication were successful (n = 2 or 4 as stated in the manuscript).
Randomization	Randomization was not relevant. All the biochemical reactions were conducted on the same 384-well plate using Echo liquidhander at the same time. No biological samples were used when conducting the assays.
Blinding	Blinding was not relevant. All the biochemical reactions were conducted on the same 384-well plate using Echo liquidhander at the same time. All the data were processed using the same computational procedure.

Reporting for specific materials, systems and methods

We require information from authors about some types of materials, experimental systems and methods used in many studies. Here, indicate whether each material, system or method listed is relevant to your study. If you are not sure if a list item applies to your research, read the appropriate section before selecting a response.

Materials & experimental systems

n/a	Involved in the study	
☒	☐	Antibodies
☒	☐	Eukaryotic cell lines
☒	☐	Palaeontology and archaeology
☒	☐	Animals and other organisms
☒	☐	Human research participants
☒	☐	Clinical data
☒	☐	Dual use research of concern

Methods

n/a	Involved in the study	
☒	☐	ChIP-seq
☒	☐	Flow cytometry
☒	☐	MRI-based neuroimaging
